# A quantum-information theoretic analysis of three-flavor neutrino oscillations: Quantum entanglement, nonlocal and nonclassical features of neutrinos

**DOI:** 10.1140/epjc/s10052-015-3717-x

**Published:** 2015-10-13

**Authors:** Subhashish Banerjee, Ashutosh Kumar Alok, R. Srikanth, Beatrix C. Hiesmayr

**Affiliations:** Indian Institute of Technology Jodhpur, Jodhpur, 342011 India; Poornaprajna Institute of Scientific Research, Sadashivnagar, Banglore, 560080 India; University of Vienna, Boltzmanngasse 5, 1090 Vienna, Austria

## Abstract

Correlations exhibited by neutrino oscillations are studied via quantum-information theoretic quantities. We show that the strongest type of entanglement, *genuine* multipartite entanglement, is persistent in the flavor changing states. We prove the existence of Bell-type nonlocal features, in both its absolute and genuine avatars. Finally, we show that a measure of nonclassicality, dissension, which is a generalization of quantum discord to the tripartite case, is nonzero for almost the entire range of time in the evolution of an initial electron-neutrino. Via these quantum-information theoretic quantities, capturing different aspects of quantum correlations, we elucidate the differences between the flavor types, shedding light on the quantum-information theoretic aspects of the weak force.

## Introduction

The study of correlations in quantum systems has a vast literature and draws its practical importance from potential applications to quantum technologies such as quantum cryptography and teleportation [1]. Recently, there has been a move toward extending these studies to systems in the domain of particle physics [2–21]. The neutrino is a particularly interesting candidate for such a study (see e.g. the review on flavor oscillation [22]). In Nature, neutrinos are available in three flavors, viz, the electron-neutrino $\nu _e$, muon-neutrino $\nu _\mu$, and tau-neutrino $\nu _\tau$. Owing to their nonzero mass, they oscillate from one flavor to another. This has been confirmed by a plethora of experiments, using both natural and “man-made” neutrinos.

Neutrino oscillations are fundamentally three-flavor oscillations. However, in some cases, they can be reduced to effective two-flavor oscillations [21]. These elementary particles interact only via weak interactions; consequently the effect of decoherence, as compared to other particles widely utilized for quantum-information processing, is small. Numerous experiments have revealed interesting details of the physics of neutrinos [23–28]. This paper asks what type of quantum correlations is persistent in the time evolution of an initial $\nu _e$ or $\nu _\mu$ or $\nu _\tau$. It presents a systematic study of the many-faceted aspect of quantum correlations. Herewith, it contributes to the understanding how Nature processes quantum information in the regime of elementary particles and, in particular, which aspect of quantum information is relevant in weak interaction processes.

Three-flavor neutrino oscillations can be studied by mapping the state of the neutrino, treating it as a three-mode system, to that of a three-qubit system [16, 17]. In particular, it was shown that the neutrino oscillations are related to the multi-mode entanglement of single-particle states which can be expressed in terms of flavor transition probabilities. Here we take the study of such foundational issues further by characterizing three-flavor neutrino oscillations by quantum correlations. This is non-trivial as quantum correlations in three-qubit systems are much more involved compared to their two-qubit counterparts.

The present study of quantum correlations in three-flavor neutrino oscillations can be broadly classified into three categories:

- Entanglement: We study various types of the in-separability properties of the dynamics of neutrino oscillations via the von Neumann entropy and in terms of a nonlinear witness of *genuine* multipartite entanglement introduced in Ref. [29].
- *Genuine* multipartite nonlocality: Nonlocality—which is considered to be the strongest manifestation of quantum correlations—is studied in both its absolute and genuine tripartite facets, characterized by the Mermin inequalities [30] and Svetlichny inequalities [31].
- Dissension: A tripartite generalization of quantum discord which is a measure of nonclassicality of correlations [32].

The plan of the paper is as follows. In Sect. 2, we provide a brief introduction to the phenomenology of neutrinos and introduce the three-flavor mode entangled state which will be analyzed using information theoretic tools. The core of the paper is Sect. 3, where we characterize three-flavor neutrino oscillations in terms of various facets of quantum correlations. We then conclude by providing an outlook.

## Three-flavor neutrino oscillations

The three flavors of neutrinos, $\nu _e$, $\nu _{\mu }$, and $\nu _{\tau }$, mix to form three mass eigenstates $\nu _1$, $\nu _2$, and $\nu _3$:

1 $$\begin{aligned} \left( \begin{array}{l} \nu _e \\ \nu _{\mu } \\ \nu _{\tau }\end{array}\right) = U \left( \begin{array}{l} \nu _1 \\ \nu _2 \\ \nu _3\end{array}\right) \,, \end{aligned}$$

where *U* is the $3\times 3$ PMNS (Pontecorvo–Maki–Nakagawa–Sakata) mixing matrix parameterized by three mixing angles ($\theta _{12}$, $\theta _{23}$, and $\theta _{13}$) and a *CP* violating phase $\delta$ (*C*...charge conjugation, *P*...parity). Neglecting the *CP* violating phase (which has not yet been observed) the mixing matrix can be written as

2 $$\begin{aligned} U = \left( \begin{array}{l@{\quad }l@{\quad }l} c_{12} c_{13} &{} s_{12} c_{13} &{} s_{13} \\ - s_{12} c_{23}- c_{12} s_{23} s_{13} &{} c_{12} c_{23}- s_{12} s_{23} s_{13} &{} s_{23} c_{13} \\ s_{12} s_{23}- c_{12} c_{23} s_{13}&{} - c_{12} s_{23}- s_{12} c_{23} s_{13} &{} c_{23} c_{13} \end{array}\right) \,,\nonumber \\ \end{aligned}$$

where $c_{ij}$ and $s_{ij}$ denote $\cos \theta _{ij}$ and $\sin \theta _{ij}$, respectively.

Therefore, each flavor state is given by a linear superposition of the mass eigenstates,

3 $$\begin{aligned} \left| \nu _{\alpha }\right\rangle = \sum _k U_{\alpha k} \left| \nu _k\right\rangle \,, \end{aligned}$$

where $\alpha = e, \mu , \tau$; $k = 1,2,3$. As the massive neutrino states $\left| \nu _k\right\rangle$ are eigenstates of the Hamiltonian with energy eigenvalues $E_k$, the time evolution of the mass eigenstates $\left| \nu _{k}\right\rangle$ is given by

4 $$\begin{aligned} \left| \nu _{k}(t)\right\rangle = e^{-\frac{i}{\hbar } E_{k} t} \left| \nu _{k}\right\rangle \,, \end{aligned}$$

where $\left| \nu _{k}\right\rangle$ are the mass eigenstates at time $t=0$.

Straightforwardly, the time evolution of flavor neutrino states is computed to be

5 $$\begin{aligned} \left| \nu _{\alpha }(t)\right\rangle = a_{\alpha e} (t)\left| \nu _e\right\rangle + a_{\alpha \mu } (t)\left| \nu _{\mu }\right\rangle + a_{\alpha \tau } (t)\left| \nu _{\tau }\right\rangle \,, \end{aligned}$$

with

6 $$\begin{aligned} a_{\alpha \beta } (t) = \sum _k U_{\alpha k}\, e^{-\frac{i}{\hbar } E_k t}\, U^{*}_{\beta k}\,. \end{aligned}$$

For example, if an electron-neutrino is produced at time $t=0$, then its time evolution is given by

7 $$\begin{aligned} \left| \nu _{e}(t)\right\rangle = a_{ee} (t)\left| \nu _e\right\rangle + a_{e\mu }(t)\left| \nu _{\mu }\right\rangle + a_{e\tau } (t)\left| \nu _{\tau }\right\rangle \,, \end{aligned}$$

where

$$\begin{aligned} a_{ee} (t)= & {} |U_{e1}|^2 e^{-\frac{i}{\hbar } E_1 t} + |U_{e2}|^2 e^{-\frac{i}{\hbar } E_2 t} + |U_{e3}|^2 e^{-\frac{i}{\hbar } E_3 t}, \\ a_{e\mu } (t)= & {} U_{e1} U_{\mu 1}^* e^{-\frac{i}{\hbar } E_1 t} \!+\! U_{e2} U_{\mu 2}^* e^{-\frac{i}{\hbar } E_2 t} \!+\! U_{e3} U_{\mu 3}^* e^{-\frac{i}{\hbar } E_3 t},\\ a_{e\tau } (t)= & {} U_{e1} U_{\tau 1}^* e^{-\frac{i}{\hbar } E_1 t} \!+\! U_{e2} U_{\tau 2}^* e^{-\frac{i}{\hbar } E_2 t} + U_{e3} U_{\tau 3}^* e^{-\frac{i}{\hbar } E_3 t}. \end{aligned}$$

If we assume that the detected neutrinos have an energy of at least 1 MeV (the electron/positron mass), namely being in the ultrarelativistic regime, the flavor eigenstates are well defined in the context of quantum mechanics [16]. In this approximation the survival probabilities take the form

8 $$\begin{aligned} P_{\nu _{\alpha } \rightarrow \nu _{\alpha }} = 1-4 \sum _{k>j} |U_{\alpha k}|^2 |U_{\alpha j}|^2 \sin ^2\left( \frac{{\varDelta } m^2_{kj} c^4}{4\hbar c }\frac{L}{E}\right) , \end{aligned}$$

and the oscillation probabilities

9 $$\begin{aligned} P_{\nu _{\alpha } \rightarrow \nu _{\beta }} = -4 \sum _{k>j} Re\{U_{\alpha k}^*U_{\beta k}U_{\alpha j} U_{\beta j}^*\}\sin ^2\left( \frac{{\varDelta } m^2_{kj} c^4}{4\hbar c }\frac{L}{E}\right) ,\nonumber \\ \end{aligned}$$

where ${\varDelta } m^2_{kj}= m^2_k -m^2_j$. As in the neutrino oscillation experiments, the known quantity is the distance *L* between the source and the detector and not the propagation time *t*; therefore the propagation time *t* is replaced by the source and detector distance *L* in the above equation. This is a valid approximation as all detected neutrinos in the oscillation experiments are ultrarelativistic.

The allowed ranges of the six oscillation parameters, three mixing angles and three mass squared differences, are obtained by a global fit to solar, atmospheric, reactor, and accelerator neutrino data within the framework of three-flavor neutrino oscillations. For normal ordering, the best fit values of the three-flavor oscillation parameters are [33]

10 $$\begin{aligned} \theta _{12} = 33.48^{\circ }, \quad \theta _{23} = 42.3^{\circ },\quad \theta _{13} = 8.50^{\circ }, \end{aligned}$$

11 $$\begin{aligned} \frac{{\varDelta } m^2_{21} c^4}{10^{-5}\, \mathrm{eV^2}} = 7.50 \,,\quad \frac{{\varDelta } m^2_{31}(\simeq {\varDelta } m^2_{32})c^4}{10^{-3} \,\mathrm{eV^2}} = 2.457 \,. \end{aligned}$$

Following Ref. [16] we introduce the occupation number of neutrinos by making the following correspondence:

12 $$\begin{aligned} \left| \nu _e\right\rangle\equiv & {} \left| 1\right\rangle _e \otimes \left| 0\right\rangle _{\mu }\otimes \left| 0\right\rangle _{\tau } \equiv \left| 100\right\rangle , \nonumber \\ \left| \nu _{\mu }\right\rangle\equiv & {} \left| 0\right\rangle _e \otimes \left| 1\right\rangle _{\mu }\otimes \left| 0\right\rangle _{\tau }\equiv \left| 010\right\rangle , \nonumber \\ \left| \nu _{\tau }\right\rangle\equiv & {} \left| 0\right\rangle _e \otimes \left| 0\right\rangle _{\mu }\otimes \left| 1\right\rangle _{\tau }\equiv \left| 001\right\rangle . \end{aligned}$$

Consequently, we can view the time evolution of a flavor eigenstate $\alpha =e,\mu ,\tau$ as a three-qubit state, i.e.,

13 $$\begin{aligned} |{\varPsi }(t)\rangle _\alpha = a_{\alpha e}(t)\;\left| 100\right\rangle +a_{\alpha \mu }(t)\;\left| 010\right\rangle +a_{\alpha \tau }(t)\;\left| 001\right\rangle . \end{aligned}$$

Therefore, flavor oscillations can be related to the time variation of the tripartite entanglement of single-particle states.

## Study of quantum-information theoretic properties in neutrino oscillations

Separability or the lack of separability, i.e., entanglement, is defined for a given state according to its possible factorization with respect to a given algebra [34]. The separability problem is in general a NP-hard problem, and only necessary but not generally sufficient criteria exist to detect entanglement. For bipartite quantum systems it suffices to ask whether the state is entangled or not. In the multipartite case the problem is more involved, since there exist different hierarchies of separability (defined later). We have defined the algebra by introducing the occupation number of the three flavors and our first goal is to understand the time evolution of neutrino oscillation in terms of tools for classifying and detecting different types of entanglement.

The next step would be to take potential measurement settings into account and analyze the different facets of the correlations in the dynamics of the neutrinos. In particular, we are interested whether there are correlations stronger than those predicted by any classical theory. The correlations are studied via two different approaches, one based on the dichotomy between predictions of quantum theory and different hidden parameter theories, and the other one quantifies the various information contents via entropies.

**Fig. 1:**
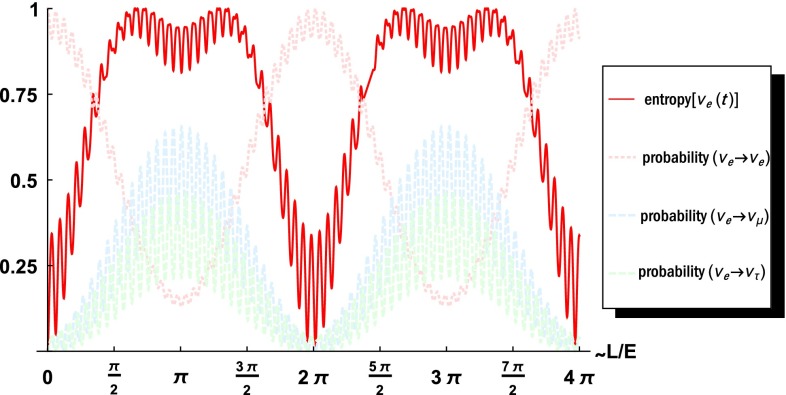
Plot of the (normalized) flavor entropy (*solid line*, *red*) and the three probabilities ($\nu _e\rightarrow \nu _e$ (*pink*, *dashed*) [survival probability, Eq. (8)], $\nu _e\rightarrow \nu _\mu$ (*light blue*, *dashed*), $\nu _e\rightarrow \nu _\tau$ (*light green*, *dashed*) [oscillation probabilities, Eq. (9)] for an initial electron-neutrino state $|{\varPsi }(t=0)\rangle _e=\nu _e(0)$ as a function of the distance traveled per energy *L* / *E*

### Study of the entanglement properties

Entanglement measures quantify how much a quantum state $\rho$ fails to be separable. Axiomatically, it must be a nonnegative real function of a state which cannot increase under local operations and classical communication (LOCC), and which is zero for separable states. An entropic function generally quantifies the average information gain by learning about the outcome obtained by measuring a system. The von Neumann entropy, a quantum mechanical analog of the Shannon entropy, is defined by $S(\rho )=-\rho \log \rho$ and is zero for pure states and $\log (d)$ for the totally mixed state, where *d* is system dimension, and the log function usually refers to base 2. The entanglement content can be computed by the entropy of the subsystems since the full system is pure.

**Fig. 2:**
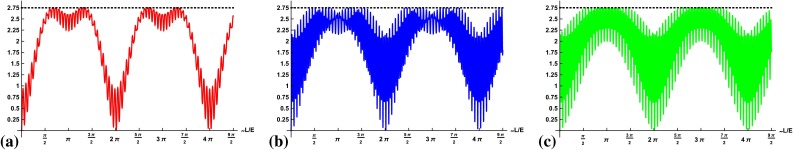
Plot of the (not-normalized) flavor entropy $S_\text {flavor}$, Eq. (14), for the three initial flavor states **a** $\nu _{e}$, **b** $\nu _{\mu }$, **c** $\nu _{\tau }$ in terms of the distance traveled per energy *L* / *E* in units of the oscillation period of the two lightest neutrinos. The *horizontal* (*dotted*) *line* corresponds to the value of the *W*-state

Considering the three possible partial traces of the three-qubit state under investigation, we obtain a concave function of the single-mode probabilities $|a_{\alpha \beta }(t)|^2$, i.e., with $\rho _j:=\text {Tr}_{\text {all but not subsystem} j}|{\varPsi }(t)\rangle _\alpha \langle {\varPsi }(t)|_\alpha$

14 $$\begin{aligned}&S_\mathrm{{flavor}}(|{\varPsi }(t)\rangle _\alpha ) = -\sum _{j=e,\mu ,\tau } \mathrm{Tr}(\rho _j \log \rho _j) \nonumber \\&\quad = - \sum _{\beta =e,\mu ,\tau } |a_{\alpha \beta }(t)|^2 \log |a_{\alpha \beta }(t)|^2\nonumber \\&\qquad - \sum _{\beta =e,\mu ,\tau } (1-|a_{\alpha \beta }(t)|^2) \log (1-|a_{\alpha \beta }(t)|^2), \end{aligned}$$

which we call the flavor entropy. This function is plotted in Fig. 1 together with the survival probabilities $\nu _e\rightarrow \nu _e$, Eq. (8), and the oscillation probabilities $\nu _e\rightarrow \nu _\mu ,\,\nu _e\rightarrow \nu _\tau$, Eq. (9).

Since the flavor entropy $S_\mathrm{{flavor}}(|{\varPsi }(t)\rangle _e)$ is nonzero for almost all time instances, the state is entangled. For this and all the following plots, we use the oscillation period of an electron- to a muon-neutrino as a unit. Since there are tiny changes in the behavior in one period due to the existence of the third flavor, we always plot two periods. When the amount of all three probabilities, both the survival as well as the oscillation probabilities, become nearly equal, the flavor entropy becomes maximal. Then the $\nu _\mu$ and $\nu _\tau$ oscillation probabilities become greater than the survival probability of $\nu _e$, resulting in a decrease in the uncertainty of the total state followed by an increase, when the probabilities get closer. Next, the uncertainty in the total state drops again and the pattern is repeated.

The entropy of all three neutrino flavors are compared in Fig. 2 showing that for the muon- and tau-neutrinos the entropy is nonzero for almost all time instances. Compared to the electron-neutrino evolution, the flavor uncertainty of the other two flavors oscillates more rapidly and with higher amplitudes, reaching the maximal value more often.

Let us now refine the picture by investigating the type of entanglement in neutrino oscillations. A tripartite pure state can, for example, be written as

15 $$\begin{aligned} |\psi _{k=3}\rangle= & {} |\phi _A\rangle \otimes |\phi _B\rangle \otimes |\phi _C\rangle ,\nonumber \\ |\psi _{k=2}\rangle= & {} |\phi _A\rangle \otimes |\phi _{BC}\rangle ,\quad |\phi _B\rangle \otimes |\phi _{AC}\rangle ,\nonumber \\&\text {or}\quad |\phi _{AB}\rangle \otimes |\phi _{C}\rangle ,\nonumber \\ |\psi _{k=1}\rangle= & {} |\psi \rangle _{ABC}, \end{aligned}$$

where *k* gives the number of partitions dubbed the *k*-separability. If *k* equals the number of involved states, in our case $k=3$, the joint state is called fully separable, else it is partially separable. An important class of states are those that are not separable within any bipartition; they are called *genuinely multipartite entangled*. In general they allow for applications that outperform their classical counterparts, such as secret sharing [35, 36]. It should be noted that since a $k=3$-separable state is necessarily also $k=2$-separable, *k*-separable states have a nested-convex structure.

Among the genuinely multipartite entangled states, there are two subclasses known for three-qubit states, the GHZ- and W-type of states. In Ref. [29] a general framework was introduced to detect and define different relevant multipartite entanglement subclasses and refined in several follow ups. In particular it has been shown to allow for a self-consistent classification also in a relativistic framework [37]. Generally, one would expect from a proper classification of different types of entanglement that for a relativistically boosted observer, which causes a change of the observed state, but not of the expectation value, it remains in a certain entanglement class. We will therefore investigate this Lorentz invariant criterion, though let us emphasize that we do not take any relativistic effects of a boosted observer into account in this contribution.

The necessary criterion for a tripartite qubit state with one excitation (“1”) to be bipartite reads

16 $$\begin{aligned}&Q_\mathrm{Dicke}^1(\rho )= 2 \left| \langle 001|\rho |010\rangle \right| +2 \left| \langle 001|\rho |100\rangle \right| +2 \left| \langle 010|\rho |100\rangle \right| \nonumber \\&\quad -\biggl (\langle 001|\rho |001\rangle +\langle 010|\rho |010\rangle +\langle 100|\rho |100\rangle \nonumber \\&\quad + 2 \sqrt{\langle 000|\rho |000\rangle \cdot \langle 011|\rho |011\rangle }+2 \sqrt{\langle 000|\rho |000\rangle \cdot \langle 101|\rho |101\rangle } \nonumber \\&\quad +2 \sqrt{\langle 000|\rho |000\rangle \cdot \langle 110|\rho |110\rangle }\biggr )\le 0. \end{aligned}$$

If this criterion is violated the state $\rho$ has no bipartite decompositions, i.e., it is genuinely multipartite entangled. The positive terms are exactly the only nonzero off-diagonal terms of the *W*-state, $|W\rangle = \frac{1}{\sqrt{3}}\lbrace |100\rangle +|010\rangle +|001\rangle \rbrace$, with one excitation in the computational basis, whereas the negative terms are only diagonal terms. Note that these negative terms are all zero for the *W*-state in the given basis such that only this state obtains the maximum value.

**Fig. 3:**
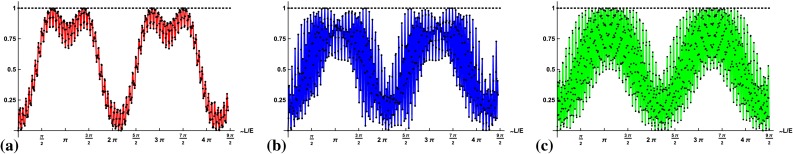
Plot of the criterion $Q_\mathrm{Dicke}^1$ detecting genuine multipartite entanglement, Eq. (16), optimized over local unities for the three initial flavor states **a** $\nu _{e}$, **b** $\nu _{\mu }$, **c** $\nu _{\tau }$ with respect to the distance traveled per energy *L* / *E* in units of the oscillation period of the two lightest neutrinos (300 data points). The criterion detects genuine multipartite entanglement if it is greater than 0 and is maximal ($=$1) only for the *W*-state

Obviously, this criterion depends on the basis representation of the state $\rho$ and has therefore to be optimized over all local unitary operations. Indeed, taking the “flavor basis” as the computational basis, Eq. (13), the unoptimized criterion becomes

17 $$\begin{aligned}&2 |a_{\alpha e}(t)a_{\alpha \mu }(t)|+2 |a_{\alpha e}(t)a_{\alpha \tau }(t)|+ 2 |a_{\alpha \mu }(t)a_{\alpha \tau }(t)|\nonumber \\&\quad -\underbrace{(|a_{\alpha e}(t)|^2+|a_{\alpha \mu }(t)|^2+|a_{\alpha \tau }(t)|^2)}_{=1}\quad \le 0, \end{aligned}$$

which is not violated for all times. Consequently, optimization over all local unitaries has to be taken into account for each time point and is plotted for an initial electron-, muon-, and tau-neutrino in Fig. 3. We find that the states at each time point are always genuine multipartite entangled if at least two amplitudes of the state, Eq. (13), are nonzero, i.e., for almost all time instances. The results depicted in Fig. 3 also prove that in the course of the time evolution the genuine multipartite *W* state (all amplitudes equal to $\frac{1}{\sqrt{3}}$) is reached. Hence, Nature exploits the maximum genuine multipartite entanglement in the occupation number basis.

**Fig. 4:**
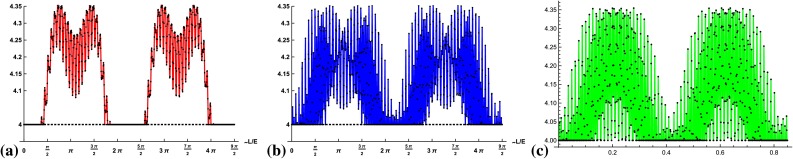
Plot of the Svetlichny criteria detecting genuine multipartite nonlocality, Eq. (19), optimized over possible bipartitions and optimized over all six different observables for the three initial flavor states **a** $\nu _{e}$, **b** $\nu _{\mu }$, **c** $\nu _{\tau }$ as a function of the distance traveled per energy *L* / *E* in units of the oscillation period of the two lightest neutrinos (300 data points). The criterion detects genuine multipartite nonlocality if the value is above 4

### Genuine multipartite mode-nonlocality

We now ask the question whether in the course of the flavor oscillations, Bell-type nonlocality, a mode-nonlocality, is persistent, i.e., there are correlations stronger than those predicted by any classical hidden variable theory. For that we investigate the Svetlichny inequalities [31] which are a sufficient criterion for proving *genuine* tripartite nonlocality. In short, the idea is whether by measuring three observables *A*, *B*, *C* and obtaining the results *a*, *b*, *c*, the probability *P*(*a*, *b*, *c*) can be assumed to be factorizable as

18 $$\begin{aligned} P(a, b, c)= & {} \int \mathsf {f}(a b|\lambda )\cdot \mathsf {h}(c|\lambda )\; \mathrm{d}\omega (\lambda ) , \end{aligned}$$

where $\mathsf {f},\mathsf {h}$ are probabilities conditioned to the hidden variable $\lambda$ with the probability measure $\mathrm{d}\omega$. The factorization, here chosen between the partitions *A*, *B* versus *C*, corresponds to Bell’s locality assumption in his original derivation if considered for two systems. [The requirement of a full factorization, i.e., the additional factorization $\mathsf {f}(a b |\lambda )= \mathsf {q}(a\lambda )\cdot \mathsf {r}(b \lambda )$, which corresponds to absolute locality, is explored later by the inequalities (20).] Then the necessary criteria for such a factorization of the conditioned probabilities are given by

19 $$\begin{aligned} I^a(\rho )= & {} \mathrm{Tr}\left( (ADC+AD'C'+A'D' C-A'D C')\rho \right) \le 4,\nonumber \\ I^b(\rho )= & {} \mathrm{Tr}\left( (AD'C+AD'C'-A'D C'-A'D'C')\rho \right) \!\le \! 4,\nonumber \\ \end{aligned}$$

with $D=B+B'$ and $D'=B-B'$. Note that we are not interested in a particular hidden parameter model, such as ($e\nu |\tau$). Consequently the above equations must be satisfied for any bipartitions, namely ($e\nu |\tau$), ($e\tau |\nu$), and ($\nu \tau |e$). In Fig. 4 we have plotted the maximum of $I^a$ and $I^b$, over all bipartitions, for the time evolution of an initial electron-, muon-, and tau-neutrino. In addition, each data point corresponds to the maximum of the optimization over all possible observables *A*, *B*, *C*. In the case of an initial electron-neutrino we find regions in the time evolution when the criterion does not detect genuine mode-nonlocality, whereas for the two other neutrino flavors we observe a stronger oscillating behavior. Summing up, whereas genuine mode-nonlocal correlation is largely present in the time evolution, there are specific time regions when it vanishes.

**Fig. 5:**
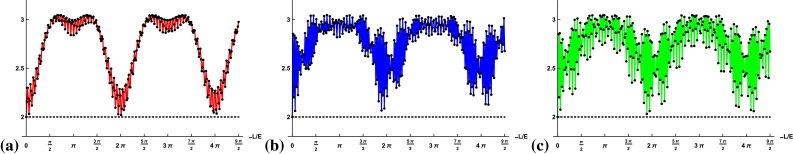
Plot of the maximum of $M^a$ and $M^b$, Eq. (20), optimized over all involved operators for the three initial flavor states **a** $\nu _{e}$, **b** $\nu _{\mu }$, **c** $\nu _{\tau }$ in terms of the distance traveled per energy *L* / *E* in units of the oscillation period of the two lightest neutrinos (300 data points). The criterion is above 2 if and only if no hidden variable model exists

Requiring that for all three measurements a hidden parameter model should exist can be revealed by the following set of inequalities [30]:

20 $$\begin{aligned} M^a(\rho )= & {} \mathrm{Tr}\left( (ADC+AD'C)\rho \right) \le 2\;,\nonumber \\ M^b(\rho )= & {} \mathrm{Tr}\left( (A'D'C-A'D C')\rho \right) \le 2\;, \end{aligned}$$

which are connected to the Svetlichny inequality by $I^a=M^a+M^b$ (see Refs. [31, 38]). These are the Mermin inequalities and their violation is an indicator of absolute nonlocality. Again we are interested in finding a contradiction to any hidden parameter model, thus we consider all bipartitions and take the maximum. The results are plotted in Fig. 5 (including an optimization over all four arbitrary operators $A,D,C,D'$). For all times (except when the state is separable) the two inequalities are violated when optimized over all measurement settings. This shows that assuming that the mode correlations can be simulated by an ensemble where all three subsystems are correlated to each other for all time instances is not possible. In contrast, correlations simulated by a hybrid mode-nonlocal–local ensemble, captured by inequalities (19), may exist for time instances close to the separable state, however, only for the electron-neutrino dynamics (Fig. 4).

It is tempting to think that this is a failure of the method, in any case we can conclude that the full time evolution of a single neutrino cannot be described by a hybrid mode-nonlocal–local ensemble for all times. Since the violation of the Svetlichny inequality is only a sufficient witness of genuine tripartite nonlocality, but not a necessary condition, it is in principle possible that the time-window where the inequality is satisfied may indeed contain this form of strong nonlocality. In any case, it seems safe to say that it should vanish close to the points where the neutrino state is characterized by a single flavor, and that genuine tripartite nonlocality is likely to be absent even in regions where genuine tripartite entanglement and absolute nonlocality may be present.

To sum up, except for small time regions, neutrino oscillations exhibit all the strong correlations, entanglement, and Bell-type mode-nonlocality that are considered to give an advantage to quantum theory over classical theories for a number of information processing tasks. For completeness, in the next section we investigate the behavior of a measure of nonclassicality weaker than entanglement.

**Fig. 6:**
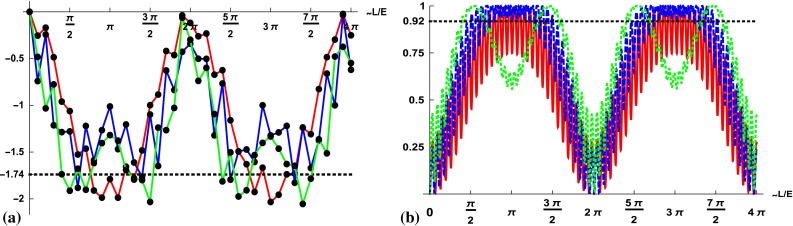
Plots of the dissensions, Eq. (24), minimized over all projective measurements for the time evolution of an initial $\nu _e$ as a function of the distance per energy *L* / *E*: **a** single-mode measure $D_1$ and **b** two-mode measure $D_2$. The *colors* encode the dependence on the reference mode: (*red* $\nu _e$), (*blue* $\nu _\mu$), (*green* $\nu _\tau$). The *horizontal lines* corresponds to the optimized values of the *W*-state, respectively. Curiously, the measures are always nonzero, detecting nonclassical correlations, and exceed the value of the *W*-state in both cases

### Dissension—a measure of nonclassicality

Classical mutual information, quantifying the information between two random variables *A* and *B*, can be defined by $I(A{:}B) = H(A) - H(A|B)$, where $H(A)=-\sum _i p_i \log p_i$ is the Shannon entropy of the probabilities *p* of the outcomes of *A* and $H(A|B):=H(A)-H(A,B)$ represents the classical conditional entropy and *H*(*A*, *B*) is the joint entropy of the pair of random variables (*A*, *B*) (see, e.g., Ref. [1]). Mutual information can be generalized for three random variables *A*, *B*, *C* by any of the following three equivalent expressions [39]:

21 $$\begin{aligned} I_1(A{:}B{:}C)= & {} H(A,B) - H(B|A) - H(A|B) -H(A|C) \nonumber \\&- H(B|C) +H(A,B|C), \nonumber \\ I_2(A{:}B{:}C)= & {} H(A) + H(B) + H(C) -H(A,B)-H(A,C)\nonumber \\&-H(B,C) + H(A,B,C), \nonumber \\ I_3(A{:}B{:}C)= & {} H(A) + H(B) + H(C) - H(A,B) \nonumber \\&- H(A,C) +H(A|B,C)\;. \end{aligned}$$

While the second of these expressions suggests a straightforward quantum generalization, by replacing the Shannon entropy by the corresponding von Neumann entropy $S(\rho ) \equiv -\text {Tr}(\rho \log \rho )$, the first and third expressions lead to complications, since the average conditioned entropy depends on the basis chosen and on the choice of the random variables *A*, *B*, *C*. Let us point out that in strong contrast to the bipartite mutual information the tripartite mutual information may also be negative. This is the case if for instance knowing the random variable *C* enhances the correlation between *A* and *B*. Following the concept of quantum discord [40, 41], which quantifies nonclassical correlations, in Ref. [32] two measures for nonclassicality, called dissension, were introduced:

22 $$\begin{aligned} D_1(A{:}B{:}C)= & {} J_1(A{:}B{:}C) - J_2(A{:}B{:}C), \nonumber \\ D_2(A{:}B{:}C)= & {} J_3(A{:}B{:}C) - J_2(A{:}B{:}C)\;, \end{aligned}$$

where the $J_i$ are the quantum analogs of the classical tripartite mutual information $I_i$, Eq. (21), namely

23 $$\begin{aligned} J_1(A{:}B{:}C)= & {} S(A,B) - S(B|{\varPi }^A) - S(A|{\varPi }^B) - S(A|{\varPi }^C) \nonumber \\&- S(B|{\varPi }^C) + S(A,B|{\varPi }^C), \nonumber \\ J_2(A{:}B{:}C)= & {} S(A) + S(B) + S(C) \nonumber \\&- S(A,B)-S(A,C)-S(B,C) + S(A,B,C), \nonumber \\ J_3(A{:}B{:}C)= & {} S(A) + S(B) + S(C) - S(A,B) - S(A,C) \nonumber \\&+ S(A|{\varPi }^{B,C})\;. \end{aligned}$$

Here $S(A|{\varPi }^B) = \sum _k p_k S(\rho _{A|{\varPi }^B_k})$ with $\rho _{A|{\varPi }^B_k} = ({\varPi }^B_k \rho _\textit{AB} {\varPi }^B_k)/p_k$ and $p_k \equiv \text {Tr}({\varPi }^B_k \rho _{AB})$ is the probability that outcome *k* is obtained. It is assumed that the basis of ${\varPi }^B_k$ is chosen such as to minimize the uncertainty. A given state is denoted to be nonclassical for any departure of $D_1$ or $D_2$ from 0. Here $D_1$ and $D_2$ deviations from zero can be associated to nonclassicality accessed by only one-mode or two-mode measurements, respectively.

We find that $J_2$ is always zero, since *S*(*A*, *B*, *C*) is zero because the total state is pure and since $S(A)=S(B,C),\;S(B)=S(A,C),\;S(C)=S(A,B)$, this being a particularity of the *W* class of states. Furthermore, any permutation of the entropy $S(A|{\varPi }^{B,C})=0$ is zero, since any projection onto the two-mode subspace gives a pure state which has zero uncertainty. Consequently, the relevant measures reduce in our case to

24 $$\begin{aligned} D_1({\varPsi }_\alpha )= & {} \bigl \lbrace S(e,\mu )-S(e,{\varPi }^\mu )-S(e,{\varPi }^\tau )-S(\mu ,{\varPi }^e)\nonumber \\&-S(\mu ,{\varPi }^\tau ),S(e,\tau )-S(e,{\varPi }^\mu )-S(e,{\varPi }^\tau )\nonumber \\&-S(\tau ,{\varPi }^e)-S(\tau ,{\varPi }^\mu ),S(\mu ,\tau )-S(\mu ,{\varPi }^e)\nonumber \\&-S(\mu ,{\varPi }^\tau )-S(\tau ,{\varPi }^e)-S(\tau ,{\varPi }^\mu )\bigr \rbrace ,\nonumber \\ D_2({\varPsi }_\alpha )= & {} \bigl \lbrace S(e),S(\mu ),S(\tau )\bigr \rbrace . \end{aligned}$$

Here the three terms in the bracket of $D_1$ refer to the single electron-neutrino, single muon-neutrino, and single tau-neutrino mode measurements, respectively. The three terms in the bracket of $D_2$ refer to joint bipartite measurements in the muon–tau, electron–tau, muon–electron mode subspaces, respectively (which are minimized to zero).

In Fig. 6 we plot the dissensions $D_1,D_2$ minimized over all projective measurements for the time evolution of an initial electron-neutrino. The first notable point is that both measures are very sensitive to whether the nonclassicality is accessed by single or bipartite measurements and both measures are nonzero for almost all times. Interestingly, we find that for both measures $\min {D_1},\min {D_2}$ and all measurement types there are time regions for which the value exceeds the corresponding value for the *W*-state, which has $(\min {D_1},\min {D_2})=(-1.738,0.918)$. For single measurements dissension $D_1$ is still considerably smaller than the values for the GHZ state ($\min {D_1}=-3$), in contrast to $D_2$ where $\min {D_2}=1$. Moreover, a strong “twin-humped” pattern of $D_2$ in the time evolution is found for joint measurements in the subspace of the two heavier neutrinos showing the existence of the third neutrino flavor ($\tau$).

## Conclusions and outlook

To sum up, we have computed several information theoretic quantities detecting and classifying correlations for the time evolution of an initial electron-, muon- or tau-neutrino. We find that for almost all time instances the neutrino states exhibit genuine quantum features.

We have analyzed in detail the dynamics of initial neutrino states via various types of entanglement properties, correlations that cannot be simulated by realistic hidden variable theories and nonclassical correlations revealed by mutual information measures. In particular, dissension turned out to be larger than that for the perfect *W*-state (Dicke state), for some time values, in strong contrast to the measures not involving measurements, i.e., the flavor entropy and the criterion detecting genuine multipartite entanglement. What physical significance this carries, if any, remains to be seen.

Qualitatively, there are differences between an initial electron-neutrino and the other two neutrinos, i.e., with the former showing less nonclassical features when compared to its heavier counterparts, a point that may merit further scrutiny. In detail we have shown that even though a genuine mode-nonlocal correlation is usually present, there are specific time regions when it vanishes. This could be described as a possible failure of the method. In any case we have proven that for the full time evolution no hybrid mode-nonlocal–local theory can be constructed.

Summing up, we can conclude that foundational issues are more prominent in accelerator experiments (mainly producing muon-neutrinos) than in reactor experiments (mainly producing electron-neutrinos).

The weak force, being one of the four known fundamental forces in Nature, dominant in the flavor changing process of neutrinos, reveals strong genuine quantum features such as also shown for weakly decaying spinless *K*-mesons [42] or for the weakly decaying half integer spin hyperons [43]. The next step would be to understand how and whether Nature takes advantage of these strong quantum correlations for information processing in a natural setting.
